# Photopigment Bleaching Phenomenon on Fluorescein Angiography in a Patient with Impending Central Retinal Vein Occlusion

**DOI:** 10.18502/jovr.v16i2.9093

**Published:** 2021-04-29

**Authors:** Narges Hassanpoor, Ahmad Mirshahi, Mohammad Reza Niyousha

**Affiliations:** ^1^Retina & Vitreous Service, Eye Research Center, Farabi Eye Hospital, Tehran University of Medical Sciences, Tehran, Iran; ^2^Retina & Vitreous Service, Nikookari Eye Hospital, Tabriz University of Medical Sciences, Tabriz, Iran

**Keywords:** Autofluorescence, Central Retinal Vein Occlusion, Fluorescein Angiography, Photopigment Bleaching, Scanning Laser Ophthalmoscopy

## Abstract

**Purpose:**

To present the second case of photopigment bleaching phenomenon in fluorescein angiography (FA) and the first case of this phenomenon due to impending central retinal vein occlusion (CRVO).

**Case Report:**

A 32-year-old healthy female noticed blurred vision in her right eye one day before presentation. Despite the 20/20 visual acuity at presentation, mild increased retinal vascular tortuosity and unilateral photopigment bleaching phenomenon in FA was observed in the right eye. Three weeks later, she developed a complete CRVO with visual acuity reduction to 20/40 that responded well to the intravitreal injection of aflibercept.

**Conclusion:**

Impending CRVO can cause unilateral photopigment bleaching phenomenon in FA that may be due to retinal ischemia.

##  INTRODUCTION 

Rhodopsin is one of the visual pigments that are responsible for absorption of the excitation beam in fundus auto-fluorescence (FAF) and fluorescein angiography (FA). After continued exposure to short- wavelength light, photo-isomerization and saturation of the visual pigments can cause decrease in their absorptive capabilities and progressive increase in background auto-fluorescence. This phenomenon is called photopigment bleaching.^[1,2]^ Similar increased background auto-fluorescence can be seen in patients with atrophic photoreceptors and retinal disorders involving the outer retina such as retinitis pigmentosa (RP), old central serous chorioretinopathy (CSCR), reattached old retinal detachment, and multiple evanescent white dot syndrome (MEWDS).^[2,3,4]^

**Figure 1:**
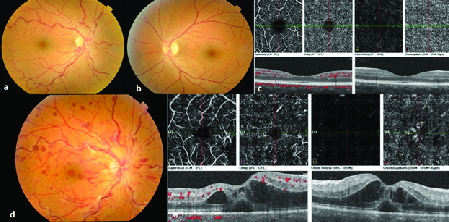
Fundus photos of the right (a) and left (b) eyes at presentation. Right eye funduscopy shows an increased vascular tortuosity and retinal dot and blot and flame shape hemorrhages that is compatible with an impending central retinal vein occlusion (CRVO). The normal optical coherence tomography angiography (OCTA) at presentation (c). After three weeks of follow up, her fundus photo was compatible with CRVO diagnosis with significantly increased vascular tortuosity and retinal hemorrhages (d). Her OCTA showed obvious macular edema and sub-retinal fluid in fovea (e).

**Figure 2:**
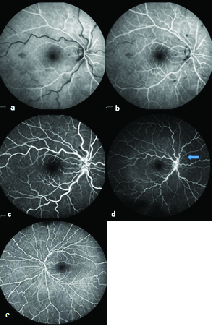
Arterial (a), lamellar (b), and arterio–venous (c) phases of fluorescein angiography (FA) with 55-degree lens shows increased vascular tortuosity and blockage areas due to retinal hemorrhages. Blue arrow: “photopigment bleaching phenomenon” (d). In previously illuminated 55-degree area, hyper-fluorescence area is seen that is surrounded with an unbleached hypo-fluorescent area in 102-degree image. The normal fellow eye 102-degree image at the same time as seen in (d) in which “photopigment bleaching phenomenon” is not visible (e).

This phenomenon was previously described in FAF.^[3,4]^ However, Breazzano et al recently reported the first case of this phenomenon in a scanning laser ophthalmoscopy (SLO)-based FA image.^[2]^ Here, we report the second case of photopigment bleaching in FA and the first case of the occurrence of this phenomenon in a case of impending central retinal vein occlusion (CRVO).

##  CASE REPORT

A 32-year-old healthy female was referred with a complaint of blurred vision in her right eye since one day before presentation. The best-corrected visual acuity (BCVA) was 20/20 in both eyes. There was a suspicious relative afferent pupil defect (RAPD) in her right eye. Results of anterior segment examination was unremarkable. The family history and her past medical history were negative. In funduscopy of the right eye, there were an increased vascular tortuosity and retinal dot and blot and flame shape hemorrhages [Figure 1]. Systemic work up was performed for hypertension, cardiac anomalies, diabetes, and hypercoagulability state that were all negative. FA was performed with confocal scanning laser ophthalmoscope system using the Heidelberg retina angiograph (Heidelberg Engineering, Carlsbad, CA). Photopigment bleaching phenomenon was seen in the late-phase FA [Figure 2d]. We reinvestigated the frame numbers taken from each eye and their time provided at the top of the print outs to rule out significant difference in number of frames taken or time of blue-light exposure in eyes. Three weeks later, she came back with visual acuity reduction to 20/40 in her right eye. Vascular tortuosity and retinal hemorrhages significantly increased in the right eye funduscopy [Figure 1d]. OCTA (Optovue, CA, USA) showed subretinal fluid and intraretinal cystic spaces and spongy edema in the foveal region [Figure 1e]. After an intravitreal injection of 2 mg /0.05 ml aflibercept (Eylea, Bayer, Germany), dramatic improvement in visual acuity was observed.

##  DISCUSSION

In FA, earlier phases images are sometimes acquired with 30- or 55-degree lenses and later images are captured with 102-degree lenses. In this situation, when exposure to short wavelength is continued or decreased tolerance of photopigments is present (e.g., due to retinal ischemia in this case), the “photopigment bleaching phenomenon” may be observed. Due to photo-isomerization and saturation of visual pigments in previously illuminated 55-degree area, we have hyper-fluorescence in central 55 degree (in round or rectangular shape which is dependent on the lens type) that is surrounded with an unbleached hypo-fluorescent area in 102-degree images [Figure 2d]. Similar increased background auto-fluorescence can be seen in patients with atrophic photoreceptors and retinal disorders that involve the outer retina.^[2,3,4]^ However, the term “photopigment bleaching phenomenon” should be used when there is not any atrophic photoreceptor layer and the increased autofluorescence is due to saturation of photopigments in the presence of structurally normal photoreceptors. In this case we did not observe any structural abnormality in macular OCT. However, mild ischemia and stasis in vascular system due to impending CRVO can cause earlier saturation of visual pigments and result in decrease of their absorptive capabilities. Unilaterality of the phenomenon in this case can be a reason for the possible effect of ischemia in this phenomenon.

##  Financial Support and Sponsorship

Nil.

##  Conflicts of Interest

There are no conflicts of interest.
